# Genetic screening for Niemann–Pick disease type C in adults with neurological and psychiatric symptoms: findings from the ZOOM study

**DOI:** 10.1093/hmg/ddt284

**Published:** 2013-06-16

**Authors:** Peter Bauer, David J. Balding, Hans H. Klünemann, David E. J. Linden, Daniel S. Ory, Mercè Pineda, Josef Priller, Frederic Sedel, Audrey Muller, Harbajan Chadha-Boreham, Richard W. D. Welford, Daniel S. Strasser, Marc C. Patterson

**Affiliations:** 1 Institute of Medical Genetics and Applied Genomics, Tübingen University, Tübingen, Germany; 2 Institute of Genetics, University College London, London, UK; 3 Regensburg University Hospital for Psychiatry and Psychotherapy, Regensburg, Germany; 4 Psychological Medicine and Clinical Neurosciences, Cardiff University, Cardiff, UK; 5 Washington School of Medicine, St Louis, MO, USA; 6 Fundació Hospital Sant Joan de Déu, CIBERER, Barcelona, Spain; 7 Department of Neuropsychiatry, Charité-Universitätsmedizin, Berlin, Germany; 8 Pitié Salpêtrière Hospital, Paris, France; 9 Actelion Pharmaceuticals Ltd, Allschwil, Switzerland; 10 Mayo Clinic, Rochester, MN, USA

## Abstract

Niemann–Pick disease type C (NP-C) is a rare, autosomal-recessive, progressive neurological disease caused by mutations in either the *NPC1* gene (in 95% of cases) or the *NPC2* gene. This observational, multicentre genetic screening study evaluated the frequency and phenotypes of NP-C in consecutive adult patients with neurological and psychiatric symptoms. Diagnostic testing for NP-C involved *NPC1* and *NPC2* exonic gene sequencing and gene dosage analysis. When available, results of filipin staining, plasma cholestane-3β,5α,6β-triol assays and measurements of relevant sphingolipids were also collected. *NPC1* and *NPC2* gene sequencing was completed in 250/256 patients from 30 psychiatric and neurological reference centres across the EU and USA [median (range) age 38 (18–90) years]. Three patients had a confirmed diagnosis of NP-C; two based on gene sequencing alone (two known causal disease alleles) and one based on gene sequencing and positive filipin staining. A further 12 patients displayed either single mutant NP-C alleles (8 with *NPC1* mutations and 3 with *NPC2* mutations) or a known causal disease mutation and an unclassified *NPC1* allele variant (1 patient). Notably, high plasma cholestane-3β,5α,6β-triol levels were observed for all NP-C cases (*n* = 3). Overall, the frequency of NP-C patients in this study [1.2% (95% CI; 0.3%, 3.5%)] suggests that there may be an underdiagnosed pool of NP-C patients among adults who share common neurological and psychiatric symptoms.

## INTRODUCTION

Niemann–Pick disease type C (NP-C) is a rare inherited lysosomal storage disorder caused by autosomal recessive mutations in either the *NPC1* gene (in 95% of cases) or the *NPC2* gene, and is characterized by progressive neurological deterioration and premature death (1,2). The incidence of NP-C is estimated at 1:120 000 live births (1,2), but this is likely an underestimate as NP-C is often misdiagnosed or detected after considerable delay (3).

The diagnosis of NP-C remains challenging due to the heterogeneous and non-specific nature of the clinical signs and symptoms. Some neurological disease manifestations such as vertical supranuclear gaze palsy (VSGP), cerebellar ataxia and gelastic cataplexy are strongly suggestive of NP-C (3–6). However, many common findings in NP-C (e.g. unexplained neonatal jaundice or isolated splenomegaly, dysarthria, dysphagia and dystonia) are shared with other conditions that involve progressive neurological deterioration.

Despite increasing knowledge of the genetics and biochemistry of NP-C, convenient, easily applied diagnostic methods are lacking. The filipin staining test has traditionally been used as the standard for diagnosis of patients with NP-C. This biochemical test involves culturing patient fibroblasts in lipoprotein-deficient serum and measuring the accumulation of unesterified cholesterol using fluorescence microscopy after LDL loading (2). However, filipin staining is both time-consuming and expensive. Complete exon sequencing of *NPC1* and *NPC2* genes, including gene dosage analysis, has increasingly been used to complement routine diagnostic tests, and more and more patients are now primarily identified by molecular genetic testing (3). As with biochemical testing, genetic tests may be inconclusive, resulting in an ‘uncertain' diagnosis.

Faster, cheaper alternatives that offer the potential to improve NP-C diagnosis are required. Emerging evidence suggests that certain cholesterol oxidation products (i.e. non-enzymatically derived oxysterols) may be useful as biochemical screening and/or disease-monitoring biomarkers (7–9). In addition, a number of recent publications have proposed plasma lysosphingolipids as potential diagnostic markers for analogous accumulated sphingolipids in a range of lysosomal storage disorders (10–13).

Patients' age at onset of neurological signs and symptoms is considered to exert a strong influence on disease progression and prognosis in NP-C (1). Evidence accrued to date has led to the definition of early infantile- (at <2 years of age), late infantile- (at age 2 to <6 years), juvenile- (at age 6 to <15 years) and adolescent/adult-onset (at ≥15 years of age) forms of NP-C (1,3). Categorization of patients by age at onset of neurological manifestations has proved useful for the evaluation of disease course and responses to therapy, as well as aiding in clinical management and genetic counselling (3).

While the infantile or childhood-onset forms of NP-C represent the majority of reported cases (1,2,14,15), increasing numbers of patients are being diagnosed in adolescence and adulthood, often with early-onset cognitive deterioration and/or psychiatric signs (14–20). Early cognitive decline occurs in almost all adolescent/adult-onset patients, and typically leads to dementia characterized by a prominent dysexecutive syndrome and memory impairment (3,21,22). Psychiatric signs, usually in the form of schizophrenia-like psychosis, have been reported in up to one-third of juvenile- and adolescent/adult-onset patients (3,17,22). Under-detection of NP-C among adults with psychosis and/or early cognitive decline remains an important challenge due to the non-specificity of these neuropsychiatric signs. In cases where the diagnosis of NP-C has been delayed, psychiatric treatments have not been reported to provide lasting benefits on psychopathology, although this has not been studied systematically (3,17). Delays in diagnosis and disease-specific therapy may affect disease outcomes and overall prognosis in affected individuals because of progressive neurodegeneration (1,3,23).

An international genetic screening study, ZOOM, was conducted to evaluate the prevalence of NP-C and to describe NP-C phenotypes among adult patients with psychosis and/or early-onset progressive cognitive decline with or without additional neurological or visceral signs suggestive of NP-C.

## RESULTS

**Figure 1.**
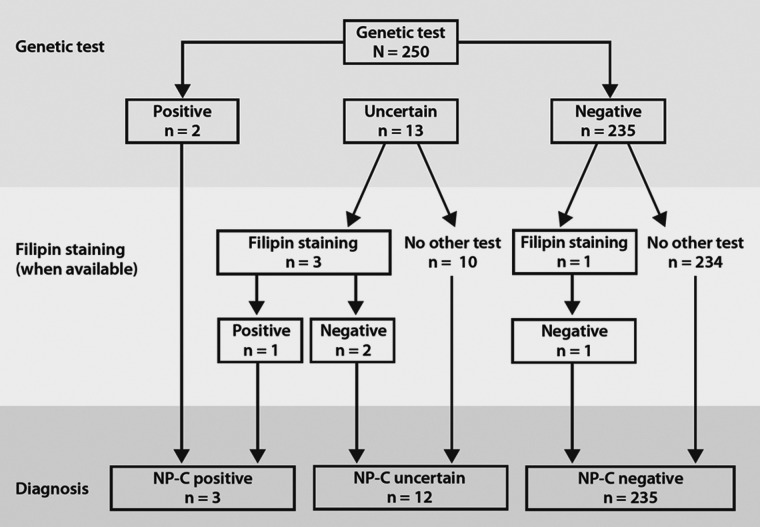
Patient flow and diagnosis.

*NPC1* and *NPC2* gene sequencing was completed in 250 of the 256 enrolled patients, who were recruited from 30 centres across the EU and USA. Figure 1 summarizes the numbers of NP-C positive (*n* = 3), NP-C uncertain (*n* = 12) and NP-C negative (*n* = 235) patients according to patient flow.

### Genetic analyses

Based on the 3/250 identified NP-C cases, the calculated frequency of NP-C in this study population was 1.2% (95% CI: 0.3%, 3.5%). Two of three NP-C positive patients (#1 and 2) were diagnosed based on gene sequencing alone and had known disease-causing gene mutations on both *NPC1* gene alleles (Table 1). The third patient (#3) had one known disease-causing mutation (c.2861C>T; p.Ser954Leu) and one unclassified *NPC1* gene variant (c.3002T>C; p.Met1001Thr), as well as a positive biochemical NP-C diagnosis based on available filipin staining results.

**Table 1.** *NPC1* and *NPC2* genotypes in ‘NP-C positive’ and ‘NP-C uncertain’ patients

Patient	NPC gene	Mutation(s)	Classification	Amino acid changes
NP-C positive patients				
1	*NPC1* *NPC1*	c.2621A>T c.2872C>T	Known NP-C mutation Known NP-C mutation	p.Asp874Val p.Arg958*
2	*NPC1* *NPC1*	c.3019C>G c.3019C>G	Known NP-C mutation Known NP-C mutation	p.Pro1007Ala p.Pro1007Ala
3	*NPC1* *NPC1*	c.2861C>T c.3002T>C	Known NP-C mutation Unclassified variant	p.Ser954Leu p.Met1001Thr
NP-C uncertain patients				
1	*NPC1*	c.563A>G	Unclassified variant	p.Asn188Ser
2	*NPC1*	c.2974G>C	Known NP-C mutation	p.Gly992Arg
3	*NPC1*	c.1712A>G	Known NP-C mutation	p.Tyr571Cys
4	*NPC2*	c.441+1G>A	Unclassified variant	NA
5	*NPC1*	c.1554–1010C>T	Unclassified variant	NA
6	*NPC1* *NPC1*	c.1990G>A c.3598A>G	Known NP-C mutation Unclassified variant	p.Val664Met p.Ser1200Gly
7	*NPC1*	c.2829C>T	Unclassified variant	p.Ile943Ile
8	*NPC1*	c.3755_3837del	Unclassified variant	p.Gly1252Alafs*56
9	*NPC2*	c.441+1G>A	Unclassified variant	NA
10	*NPC2*	c.441+1G>A	Unclassified variant	NA
11	*NPC1*	c.2861C>T	Known NP-C mutation	p.Ser954Leu
12	*NPC1*	c.2335T>C	Unclassified variant	p.Phe779Leu

Amino acid changes denoted by ‘NA’ (not applicable) indicate cases in whom splicing defects are assumed, which does not allow prediction of the amino acid change.

With 12/250 patients classified as ‘NP-C uncertain’, the study frequency of uncertain NP-C diagnoses was 4.8% (95% CI: 2.5%, 8.2%). A total of eight NP-C uncertain patients (#1, 4, 5, 7–10 and 12) displayed a mono-allelic unclassified variant, among whom five patients (#1, 5, 7, 8 and 12) had an *NPC1* variant and three (#4, 9 and 10) had an *NPC2* variant. Three patients (#2, 3 and 11) had a mono-allelic known causal disease mutation affecting the *NPC1* gene, and one patient (#6) had a known causal disease mutation and an unclassified *NPC1* allele variant (Table 1).

### Patient demographics and primary diagnosis

Table 2 summarizes the demographics of all patients according to NP-C diagnosis groups. Overall, the study included 170 male patients (68%) and 80 female patients (32%). The median (range) age was 38 (18–90) years.

**Table 2.** Demographics and patient characteristics

	NP-C positive (*n* = 3)	NP-C uncertain (*n* = 12)	NP-C negative (*n* = 235)
Gender (male:female)	1:2	8:4	161:74
Age in years, median (range)	43 (41–49)	36 (22–57)	38 (18–90)
Primary diagnosis, *n*			
Psychosis	0	9	153
Early-onset progressive cognitive decline	2	1	48
Psychosis combined with early-onset progressive cognitive decline	1	2	34
Neurological symptoms, *n*			
Present^a^	3	9	210
Focal dystonia	0	3	71
Cerebellar ataxia	2	3	66
Dysarthria	3	7	133
VSGP^b^	2	1	38
Epilepsy	0	2	65
Cataplexy	1	1	5
Absent	0	3	21
Unknown	0	0	4
Visceral symptoms, *n*			
Present^a^	1	1	32
Hepatomegaly	0	1	30
Splenomegaly	1	0	7
Absent	1	9	158
Unknown	1	2	45

^a^Multiple answers possible.

^b^Vertical supranuclear gaze palsy (positive clinical assessment at baseline visit).

All three NP-C positive patients displayed cognitive impairment (attention deficits and memory impairment) with or without concomitant psychosis. Two were assessed using the Mini-Mental State Examination (MMSE) and achieved scores of 24 and 27. All three were assessed using the verbal fluency test and scored between 3 and 14.

A large proportion (92%) of the NP-C uncertain group had psychosis, with two patients also showing concomitant early-onset progressive cognitive decline. Most commonly, psychotic disorders were diagnosed as schizophrenia, but organic psychosis with mood disorders was also recorded.

### Clinical manifestations

All three NP-C positive patients and 9/12 NP-C uncertain patients had typical neurological symptoms suggestive of NP-C (Table 2). VSGP was present in two out of three NP-C positive patients, and was also observed in 38/235 NP-C negative patients. Visceral symptoms (hepatomegaly and/or splenomegaly) were present in one out of three NP-C positive and 32/235 NP-C negative patients.

Median (range) composite disability scores were 0.29 (0.06, 0.46) in the NP-C positive group, 0.06 (0; 0.33) in the NP-C uncertain group and 0.06 (0; 1.0) in the NP-C negative group. A categorical analysis of disability scores for each of the scale domains is summarized in Supplementary Material, Table S1.

### Biochemical analysis

Filipin staining results were available for four patients. One patient had positive findings and was confirmed as NP-C positive. Negative filipin staining findings were obtained in two NP-C uncertain patients and one NP-C negative patient.

**Figure 2.**
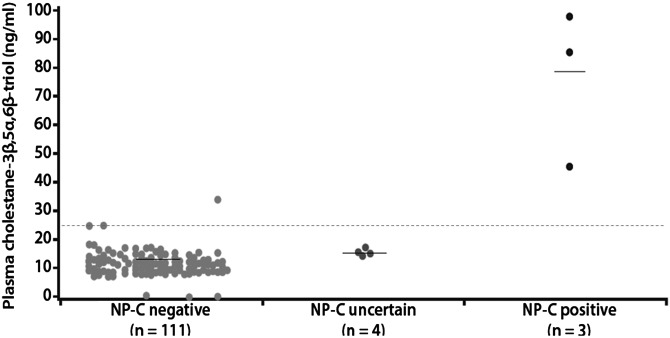
Plasma cholestane-3β,5α,6β-triol levels according to NP-C diagnosis. Horizontal dashed line denotes the putative cut-off value (24.5 ng/ml).

Plasma cholestane-3β,5α,6β-triol concentrations were measured in patients with available samples (*n* = 118), and the results are shown in Figure 2. Median (range) concentrations were 85.4 (46.1–98.7) ng/ml in NP-C positive patients (*n* = 3), 15.3 (12.7–17.3) ng/ml in NP-C uncertain patients (*n* = 4) and 11.1 (0.1–33.6) ng/ml in NP-C negative patients (*n* = 111). The three NP-C positive patients had concentrations above the cut-off value of 24.5 ng/ml. A fourth patient who was categorized as NP-C negative also had a level well above this threshold.

Among patients with available sphingolipid assessments, median (range) plasma levels of SPC were 3.8 (3.7–7.7) nm in the NP-C positive group (*n* = 3), 1.3 (1.0–1.4) nm in the NP-C uncertain group (*n* = 4) and 1.2 (0.5–10.2) nm in the NP-C negative group (*n* = 111) (Supplementary Material, Fig. S1). Median (range) plasma glucosylsphingosine levels were 6.8 (3.3–8.1) nm in the NP-C positive group (*n* = 3), 2.3 (1.2–2.4) nm in the NP-C uncertain group (*n* = 3) and 2.0 (0.3–13.9) nm in the NP-C negative group (*n* = 110) (Supplementary Material, Fig. S2). Plasma values for all eight measured sphingolipids are summarized in Supplementary Material, Table S2.

### *Post hoc* NP-C suspicion index analysis

Overall, 89% of enrolled patients had a high or moderate risk prediction score following clinical screening based on the NP-C Suspicion Index (SI). All three NP-C positive patients had a high suspicion of NP-C (risk prediction score ≥70). In the NP-C uncertain and NP-C negative groups, high proportions of patients [9/12 (75%) and 140/235 (60%), respectively] were categorized as having a moderate suspicion of NP-C (risk prediction score 40–69). Supplementary Material, Table S3 summarizes SI assessments by the NP-C diagnosis group.

## DISCUSSION

Three patients with early-onset progressive cognitive decline with or without concomitant psychosis were identified as NP-C positive from this study population based on genetic testing and available biochemical analysis by filipin staining. A novel disease-causing *NPC1* gene mutation (c.3002T>C, p.Met1001Thr) was validated by filipin staining.

The observed frequency of NP-C (1.2%; 95% CI 0.3%, 3.5%) in this study population was substantially higher than the previously reported incidence in the general population (1/120 000 live births) (1,2). Because an early onset of neurological manifestations in NP-C is associated with premature death, the frequency of NP-C among adults should actually be lower than the live birth incidence. The inclusion criteria for this study population therefore clearly resulted in an enrichment for NP-C cases.

For 12 patients (∼5% of the study population), the diagnosis remained uncertain. Further research is needed to ascertain whether these patients would be best categorized as ‘NP-C positive’ or ‘NP-C negative’.

The frequency of *NPC1* or *NPC2* gene variants observed in this study (4.8%) was higher than the frequency of heterozygous *NPC1* and *NPC2* gene variants (VUS3s) reported in the Exome Variant Project (EVP; 2.0%) (24). This further suggests an enrichment of NP-C variants in the ZOOM population. However, this comparison should be interpreted with caution as it assumes that data from whole-exome sequencing, as applied in the EVP, provides a level of sensitivity for the detection of regulatory and splice-site mutations that is comparable with the sequencing techniques employed in the current study [Sanger sequencing with Multiplex Ligation-dependent Probe Amplification (MLPA) ‘gene dosage’ analysis].

A solid interpretation of the apparent enrichment for heterozygotes in the ZOOM population must address whether it is due to: (i) insensitivity of the genetic analysis, disabling the detection of a second *NPC1* or *NPC2* mutation among NP-C uncertain patients; (ii) an unexplained bias in study recruitment that, by chance, led to inclusion of more *NPC1* and *NPC2* carriers than in the normal population; or (iii) the potential detection of NP-C patients with late-onset disease carrying only one *NPC1* or *NPC2* mutation (i.e. true heterozygotes), in whom NP-C manifests as a dominant condition with reduced penetrance.

With respect to diagnostic sensitivity, it is important to note that the genetic techniques used in the ZOOM study, although following diagnostic routine, have a number of limitations. Standard gene sequencing techniques did not cover non-coding *NPC1* or *NPC2* gene regions, and therefore might not have detected regulatory and deep intronic splicing mutations. It is also possible that some novel mutant NP-C alleles might not yet have been characterized, and therefore may be present (but go undetected) among NP-C uncertain or even NP-C negative patients. The heterozygous deletion of exon 25, observed in one ‘NP-C uncertain’ patient (#8), has not previously been published and could not be further validated by the methodology used in this study.

Secondly, regarding a potential selection bias during recruitment, NP-C uncertain patients were detected across a range of geographic regions, including the USA (two African-Americans and one Caucasian individual), Germany, France, the Czech Republic, Greece and Italy. This would argue against there having been any regional confounding effects in the ZOOM population.

Thirdly, as the ZOOM study cohort is by far the oldest and largest ever studied, it is an attractive speculation that heterozygous *NPC1* and *NPC2* might even have a dominant effect with reduced penetrance. However, there is no strong evidence in the literature to date that would indicate whether *NPC1* mutation heterozygotes can be symptomatic, and we can really only speculate on this.

Our data and other published evidence do not allow any firm conclusions on the likelihood of these intriguing possibilities. We therefore suggest further genetic analyses in NP-C uncertain patients in the future. For example, *NPC1* and *NPC2* mRNA abnormalities should be analysed to establish whether regulatory disturbances and/or cryptic splice mutations might be present. Moreover, genomic sequencing of the complete NP-C loci, including relevant upstream and downstream moieties, should allow detection of genomic variation related to pathogenic mRNA expression and processing, respectively.

Evidence from animal studies and clinical analyses have indicated that cholestane-3β,5α,6β-triol may be a promising candidate as a biomarker for NP-C. This oxysterol has previously been shown to demonstrate excellent sensitivity and specificity in the detection and monitoring of NP-C in murine and feline models (7). It reliably detects NP-C1 patients and differentiates heterozygote mutation carriers from controls (8).

While there was considerable overlap in the levels of cholestane-3β,5α,6β-triol between the NP-C uncertain and NP-C negative groups in the current study, plasma concentrations of this putative biomarker in the NP-C positive patients, including the patient with a novel *NPC1* mutation, were substantially higher. The median concentration of cholestane-3β,5α,6β-triol in the NP-C positive group was ∼7.5-fold greater than that in the NP-C negative group. Further, plasma concentrations in all three NP-C positive patients were well above the cut-off value of 24.5 ng/ml, which has previously been shown to define the upper limit of normal based on the fact that no control patients have been observed to exceed this level, and that only 2.7% of NP-C1 patients tested to date had levels below it (8). Thus, plasma cholestane-3β,5α,6β-triol was able to differentiate NP-C1 positive patients with 100% sensitivity.

With respect to the specificity of cholestane-3β,5α,6β-triol testing, three out of four patients (75%) with elevated plasma concentrations were diagnosed with NP-C. The fourth patient had a concentration of 33.5 ng/ml but did not display potential NP-C mutations, and was therefore classified as NP-C negative in accordance with the study protocol. Since *NPC1* mutation carriers can exhibit elevated plasma cholestane-3β,5α,6β-triol concentrations of between 24.5 and 38 ng/ml, we cannot discriminate whether this fourth patient is an NP-C carrier or an NP-C patient with negative genetic sequencing. Established disease guidelines specify that ambiguous test results should be worked up with further tests such as mRNA analyses, complete gene sequencing or filipin staining (3), none of which have been performed in this case.

Sphingolipids have also been proposed as candidate NP-C biomarkers. Among those measured during the exploratory sphingolipid analysis in this study, SPC and glucosylsphingosine appeared to be elevated in the NP-C positive cases compared with the total study population. Glucosylceramide and sphingomyelin are known to accumulate in the viscera in NP-C (2), and it is considered likely that plasma SPC and glucosylsphingosine could act as surrogates for the accumulation of their respective analogous N-acylated sphingolipids. To our knowledge, this is the first time that plasma SPC and glucosylsphingosine have been assessed as potential diagnostic biomarkers for NP-C. Further NP-C cohort studies incorporating age-matched controls are required in order to establish their sensitivity and specificity, and to understand how they might relate to NP-C in terms of disease onset and severity.

Plasma oxysterols and sphingolipids were assessed according to a late protocol amendment (without any changes to the inclusion criteria). As a result, these analytes were only quantified in half of the study population, and the low number of measurements in the NP-C positive and NP-C uncertain groups did not allow calculation of useful measures of variation, for discriminatory purposes. It is considered unlikely that this limitation was the source of any significant selection bias, due to the nature of the protocol amendment. The biomarker data are therefore considered representative of the entire ZOOM study population.

The *post hoc* clinical screening based on the NP-C SI did not yield much additional information in this study because most disease features comprising the NP-C SI itself were reflected in the ZOOM study inclusion criteria. Patients diagnosed with psychosis or early onset progressive cognitive decline and suggestive neurological symptoms therefore score highly on the SI. The proportion of patients with moderate or high scores on the SI across the diagnostic groups in this study was between 83 and 100%, which is appreciably higher than the proportion of patients scoring at this level in the much broader patient population on which the NP-C SI developmental study was based (6). In addition, the NP-C SI threshold for high suspicion was defined for screening purposes in unselected populations, and therefore its poor predictive value in a selected adult patient sample such as that in ZOOM is not surprising (4).

A remarkably large number of patients in the NP-C negative group in the current study had VSGP, which is associated with a high score weighting within the SI, accounting for the relatively high proportion of NP-C negative patients with high SI scores. It would be of interest to determine the final diagnoses in these patients.

The data on the frequency of VSGP should be interpreted with caution. Considering the study inclusion criteria, the study population could possibly have been enriched for other neurological disorders involving oculomotor impairment, such as progressive supranuclear palsy, but no differential diagnosis data were collected. In addition, only the presence or absence of VSGP was recorded on the study CRF for each patient—no further information as to the degree of oculomotor impairment was collected. Given the multicentre nature of the study, with multiple clinicians conducting assessments without the aid of standard equipment, it is possible that VSGP was reported in patients who actually showed lesser degrees of impairment (e.g. delayed saccadic initiation and slowed saccades).

In conclusion, it remains difficult to establish a diagnosis of NP-C owing to the protean clinical manifestations associated with the disease, and to the technical challenges inherent in the currently available diagnostic tests. The relatively high frequency of NP-C diagnoses in this study compared with the estimated live-birth incidence in the general population suggests that there may be an underdiagnosed pool of NP-C patients present among adults who share common neurological and psychiatric symptoms. Recognizing the appropriate combination of clinical manifestations, genetic testing and biochemical analyses could help improve NP-C diagnosis in this selected population.

## MATERIALS AND METHODS

### Patients

Consecutive patients aged ≥18 years and undergoing medical care in psychiatric or neurological reference centres across the EU and USA were included between 15 September 2010 and 18 November 2011. Patients were required to meet at least one of the selection criteria listed in Table 3. Patients with a previous diagnosis of a lipid storage disorder (including NP-C), or with neurological symptoms, psychosis or early-onset progressive cognitive decline clearly related to hereditary or acquired disease other than NP-C, or to drugs such as neuroleptics, were excluded.

**Table 3.** Patient selection criteria

Patients falling in one of the following categories	
A	Psychosis^a^ combined with ≥1 pre-defined neurological^^b^^ or visceral^^c^^ symptom/syndrome
B	Early-onset progressive cognitive decline^^d^^ combined with ≥1 pre-defined neurological^^b^^ or visceral^^c^^ symptom/syndrome
C	Early-onset progressive cognitive decline^^d^^ combined with psychosis^a^

^a^Psychosis defined according to ICD-10 and DSM-IV criteria, with onset at <50 years of age.

^b^Includes focal dystonia, cerebellar ataxia, dysarthria, vertical supranuclear gaze palsy, cataplexy, epilepsy.

^c^Hepatomegaly or splenomegaly.

^d^Early-onset progressive cognitive decline (at <50 years of age) includes dysexecutive syndrome, attention deficits, aphasia, apraxia, agnosia, memory impairment, dementia.

This study was conducted in full compliance with the principles of the ‘Declaration of Helsinki’ and its amendments. The protocol and all materials provided to the patient were reviewed and approved by appropriate Independent Ethics Committees (IECs) before study commencement at each participating centre. Written informed consent was obtained from each individual participating in the study or by their legal guardian (authorized by the respective IEC).

A multidisciplinary Independent Scientific Committee was appointed to oversee all aspects of the study conduct and data analysis. The committee's responsibilities covered review of data entry/integrity, patient diagnostic classification and data interpretation.

### Study design

This was an observational, multicentre screening study that was designed to include a cross-sectional analysis and a case–control analysis. The cross-sectional analysis evaluated *NPC1*/*NPC2* mutation carrier prevalence among the selected study population, and explored possible genotype–phenotype associations. Plasma oxysterol and sphingolipid levels were measured and compared with NP-C status in order to assess their use as potential biomarkers for NP-C. The case–control analysis was planned to assess risk predictors of NP-C in the event that sufficient numbers of patients were identified to allow a comparison of: (i) all patients diagnosed with NP-C either based on positive genetic results alone or a combination of an uncertain genetic result and a positive filipin staining result (cases) with (ii) a sub-sample of study subjects who did not have NP-C (controls).

### Data collection

Anonymized data were collected by site investigators or clinical research staff using a standard electronic data capture (EDC) form for each patient, and uploaded to a secure study database. Data quality was maintained and checked periodically, and the EDC system incorporated an audit trail for all entries and changes.

The following data were collected at the ‘baseline’ visit for all recruited patients: demographics, neurological impairment assessed by the NP-C disability scale, verbal fluency tests (22) and neurological examination.

The following retrospective data were also collected based on medical chart information for all patients diagnosed with NP-C (‘NP-C positive’ patients): psychiatric history, history of neurological signs and symptoms, history of liver disease, childhood development, neonatal medical history, ongoing and previous medication, cognitive impairment based on the MMSE (25) and neuroimaging data.

### Genetic and biochemical analyses

Blood samples were collected from all patients for *NPC1* and *NPC2* gene sequencing analysis, which included characterisation of exons and exon–intron boundaries. MLPA was performed where *NPC1* and *NPC2* sequencing results were not conclusive. Investigators were also encouraged to refer patients with uncertain NP-C gene sequencing results to NP-C reference centres for further standard work up, including filipin staining (3). When available, filipin staining results were collected in the study database.

Carriers of two known or novel mutant NP-C alleles with a published or predicted (in the case of novel mutant NP-C alleles) functional impact were diagnosed with NP-C (i.e. ‘NP-C positive’ patients). Individuals with novel mutant NP-C alleles and positive filipin staining evidence of NP-C were also diagnosed as NP-C positive. All heterozygote carriers with only one published or novel mutant NP-C allele and no filipin staining data were classified as ‘NP-C uncertain’ based on the possibility that they could either not have NP-C, or be NP-C patients in whom the molecular diagnosis had not been established due to a lack of testing sensitivity.

Plasma levels of cholestane-3β,5α,6β-triol and selected sphingolipids [sphingosyl-phosphocholine (SPC), sphingosine-1-phosphate, sphingosine, glucosylsphingosine, dihydrosphingosine-1- phosphate, dihydrosphingosine, lysoGb3 and lactosylsphingosine] were quantified by liquid chromatography-tandem mass spectrometry (LC-MS/MS) according to previously published methods in all patients with available plasma samples (8,9,26,27).

Both oxysterol and sphingolipid analyses were included via a protocol amendment, and assays were performed on anonymized samples.

### Clinical assessments

Psychiatric and clinical neurological signs and symptoms typically seen in NP-C and present at study enrolment were summarized for each patient. Psychosis was defined at enrolment according to ICD-10 and DSM-IV criteria, with onset below 50 years of age. ICD-10 categories included F06.0-3 (organic psychoses and mood disorder), F20 (schizophrenia), F22 (persistent delusional disorder), F25 (schizoaffective disorder), F30 (manic episode) and F31 (bipolar disorder). DSM-IV categories included schizophrenia (295.xx), schizoaffective disorder (295.70), psychotic disorder not otherwise specified (298.9), bipolar I disorder (296.xx) and bipolar disorder not otherwise specified (296.80).

Early-onset progressive cognitive decline (<50 years of age) was defined at enrolment as including at least one of dysexecutive syndrome, attention deficits, aphasia, apraxia, agnosia, memory impairment and/or dementia. Investigators were also encouraged to assess patients' cognitive function at enrolment based on verbal fluency tests.

Patients were specifically assessed at enrolment for the following suggestive neurological and visceral signs: VSGP, cerebellar ataxia, cataplexy, epilepsy, focal dystonia, dysarthria, hepatomegaly and splenomegaly.

Patient functional disability was evaluated at enrolment based on an NP-C-specific disability scale, which assessed neurological impairment based on four functional domains—ambulation, manipulation, language and swallowing—each rated on a four- to five-point scale with scores ranging from 0 (normal) to 1 (worse) (15,28). Deep tendon reflexes were also proactively assessed using standard clinical techniques.

In MMSE assessments, patients' cognitive function was rated on a scale from 0 [complete impairment] to 30 [no impairment]. Verbal fluency was assessed by asking patients to state all the names of fruits and vegetables that they could think of during 1 min; one point was allowed for each word (22).

Patients were assessed using an NP-C SI tool, which ranks specific symptoms within and across key disease domains, including family members who have NP-C, to provide a risk prediction score that can aid in the identification of patients who should undergo further testing for NP-C (6). The NP-C SI indicates a high suspicion of NP-C based on scores ≥70, a moderate suspicion based on scores of 40–69 and a low suspicion among patients scoring <40.

### Statistical analyses

The study protocol originally specified that 1000 patients would be recruited from 50 psychiatry/neurology reference centres. A total of 256 patients were recruited from 30 centres due to early termination of the study. As a result, only the descriptive objectives of the cross-sectional analysis could be fulfilled. Phenotype–genotype associations and analyses for risk predictors for NP-C based on case–control analyses were not conducted due to the limited numbers of cases available.

Statistical analyses were performed on the all-enrolled study population based on patients who had available diagnostic information. Descriptive statistics for demographics, plasma cholestane-3β,5α,6β-triol and plasma sphingolipid levels in these patients were calculated according to NP-C diagnosis categories (NP-C positive, NP-C uncertain and NP-C negative). Patient numbers and proportions per diagnosis category are expressed as a percentage of the number of patients with full or partial responses, with no imputation for missing values.

The NP-C disability scale result was described by frequency distribution of patients' scores for each of the four functional domains (ambulation, manipulation, language, swallowing), as well as by combined ‘composite disability score’ (15).

A *post hoc* descriptive analysis was also conducted based on patients' primary diagnosis and NP-C SI scores (6).

## SUPPLEMENTARY MATERIAL

Supplementary Material is available at *HMG* online.

## FUNDING

This study was sponsored by Actelion Pharmaceuticals Ltd. Funding to pay the Open Access publication charges for this article was provided by Actelion Pharmaceuticals Ltd.

## Supplementary Material

Supplementary Data
