# Supplementary material for: Genetic screening for Niemann–Pick disease type C in adults with neurological and psychiatric symptoms: findings from the ZOOM study
Source: Hum Mol Genet. 2013 Jun 16;22(21):4349–56. doi: 10.1093/hmg/ddt284 (PMC3792693; doi:10.1093/hmg/ddt284)
Supplement: Supplementary Data [file supp_22_21_4349__index.html]

Genetic screening for Niemann-Pick disease type C in adults with neurological and psychiatric symptoms: findings from the ZOOM study — Genetic screening for Niemann–Pick disease type C in adults with neurological and psychiatric symptoms: findings from the ZOOM study — Genetic screening for Niemann–Pick disease type C in adults with neurological and psychiatric symptoms: findings from the ZOOM study — Supplementary Data 

# Genetic screening for Niemann–Pick disease type C in adults with neurological and psychiatric symptoms: findings from the ZOOM study

## 

Supplementary Data

**Files in this Data Supplement:**

- Supplementary Data - Docx file
- Supplementary Figure 1 - tif file
- Supplementary Figure 2 - tif file
