# Supplementary material for: Genetic screening for Niemann–Pick disease type C in adults with neurological and psychiatric symptoms: findings from the ZOOM study
Source: Hum Mol Genet. 2013 Jun 16;22(21):4349–56. doi: 10.1093/hmg/ddt284 (PMC3792693; doi:10.1093/hmg/ddt284)
Supplement: Supplementary Data [file supp_ddt284_ddt284supp.docx]

**Supplementary Table 1.** NP-C disability scale categories

| **Disability scale domain** | **NP-C positive**  **(n = 3)** | **NP-C uncertain**  **(n = 12)** | **NP-C negative**  **(n = 235)** |
| --- | --- | --- | --- |
|  | **Number of patients** | | |
| **Ambulation** |  |  |  |
| Normal | 1 | 10 | 169 |
| Autonomous ataxic gait | 2 | 1 | 36 |
| Outdoor assisted ambulation | 0 | 1 | 10 |
| Indoor assisted ambulation | 0 | 0 | 8 |
| Wheelchair bound | 0 | 0 | 11 |
| **Manipulation** |  |  |  |
| Normal | 1 | 11 | 158 |
| Slight dysmetria/dystonia | 1 | 1 | 51 |
| Mild dysmetria/dystonia | 1 | 0 | 15 |
| Severe dysmetria/dystonia | 0 | 0 | 9 |
| **Language** |  |  |  |
| Normal | 0 | 4 | 82 |
| Mild dysarthria | 3 | 5 | 133 |
| Severe dysarthria | 0 | 2 | 15 |
| Non-verbal communication | 0 | 1 | 1 |
| Absence of communication | 0 | 0 | 3 |
| **Swallowing** |  |  |  |
| Normal | 1 | 11 | 200 |
| Occasional dysphagia | 1 | 1 | 22 |
| Daily dysphagia | 1 | 0 | 8 |
| NG tube or gastric button feeding | 0 | 0 | 4 |

**Supplementary Table 2.** Plasma sphingolipid levels by NP-C diagnosis

| **Sphingolipids, nM** | **NP-C positive**  **(n = 3)** | **NP-C uncertain**  **(n = 12)** | **NP-C negative**  **(n = 235)** |
| --- | --- | --- | --- |
|  | **Median (range)** | | |
| Number of patients with   sphingolipid data | 3 | 4 | 111 |
| Sphingosyl-phosphocholine (SPC) | 3.8 (3.7–7.7) | 1.3 (1.0–1.4) | 1.2 (0.5–10.2) |
| Glucosylsphingosine | 6.8 (3.3–8.1) | 2.3 (1.2–2.4) | 2.0 (0.3–13.9) |
| Sphingosine-1-phosphate | 157 (99.9–226.6) | 294 (208.2–580.5) | 188 (89.7–505.7) |
| Sphingosine | 10.7 (8.6–12.6) | 18.9 (11.5–21.1) | 11.9 (4.5–209.8) |
| Dihydrosphingosine-1-phosphate | 35.8 (19.6–39.3) | 58.9 (35.3–146.0) | 43.3 (14.8–123.8) |
| Dihydrosphingosine | 3.8 (1.9–4.3) | 4.3 (3.5–5.8) | 4.0 (1.1–53.0) |
| LysoGb3 | 4.4  (3.0–5.8) | 2.7  (0.7–3.8) | 3.1  (0.2–8.2) |
| Lactosylsphingosine* | 22,700  (15 700–32 400) | 13,800 (4 740–19 300) | 17,800 (2 470–36 000) |

**Lactosylsphingosine quantified based on peak area as unit.*

**Supplementary Table 3.** *Post hoc* analysis of SI category per NP-C diagnosis group

| **Suspicion Index category** | **NP-C positive**  **(n = 3)** | **NP-C uncertain**  **(n = 12)** | **NP-C negative**  **(n = 235)** |
| --- | --- | --- | --- |
|  | **Number of patients** | | |
| High suspicion (n = 72) | 3 | 1 | 68 |
| Moderate suspicion (n = 149) | 0 | 9 | 140 |
| Low suspicion (n = 29) | 0 | 2 | 27 |

*SI categories defined based on risk prediction scores as follows: high suspicion of NP-C (score ≥70); moderate suspicion of NP-C (score 40–69); low suspicion of NP-C (score <40).*

**SUPPLEMENTAL FIGURE TITLES**

**Supplemental Figure 1.** Plasma sphingosine phosphocholine (SPC) levels in NP-C positive, NP-C uncertain and NP-C negative patients

**Supplemental Figure 2.** Plasma glucosylsphingosine levels in NP-C positive, NP-C uncertain and NP-C negative patients
