# Supplementary figures and images for: Genetic screening for Niemann–Pick disease type C in adults with neurological and psychiatric symptoms: findings from the ZOOM study
Source: Hum Mol Genet. 2013 Jun 16;22(21):4349–56. doi: 10.1093/hmg/ddt284 (PMC3792693; doi:10.1093/hmg/ddt284)

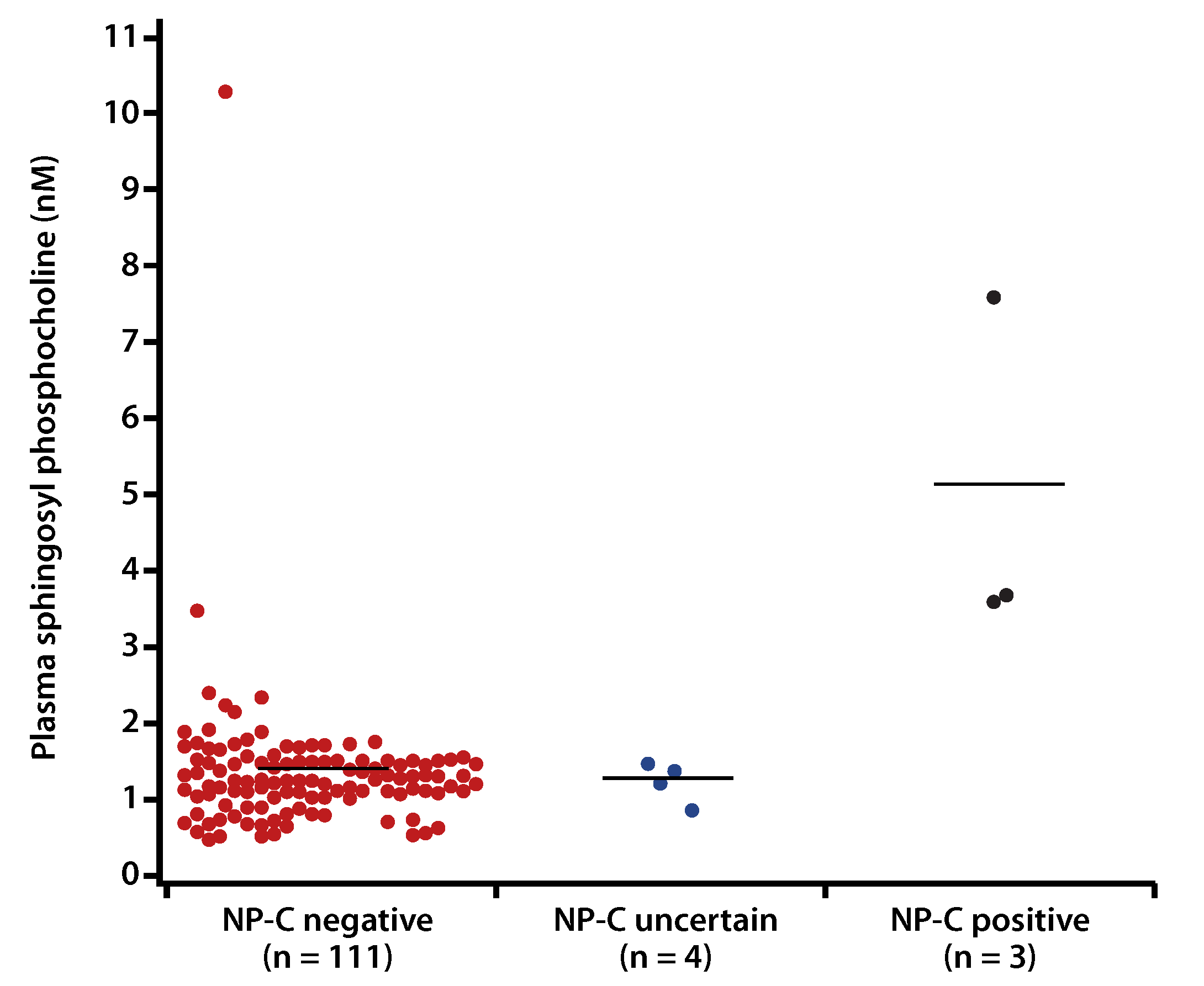

Supplement: Supplementary Data [file supp_ddt284_ddt284supp_fig1.tif]

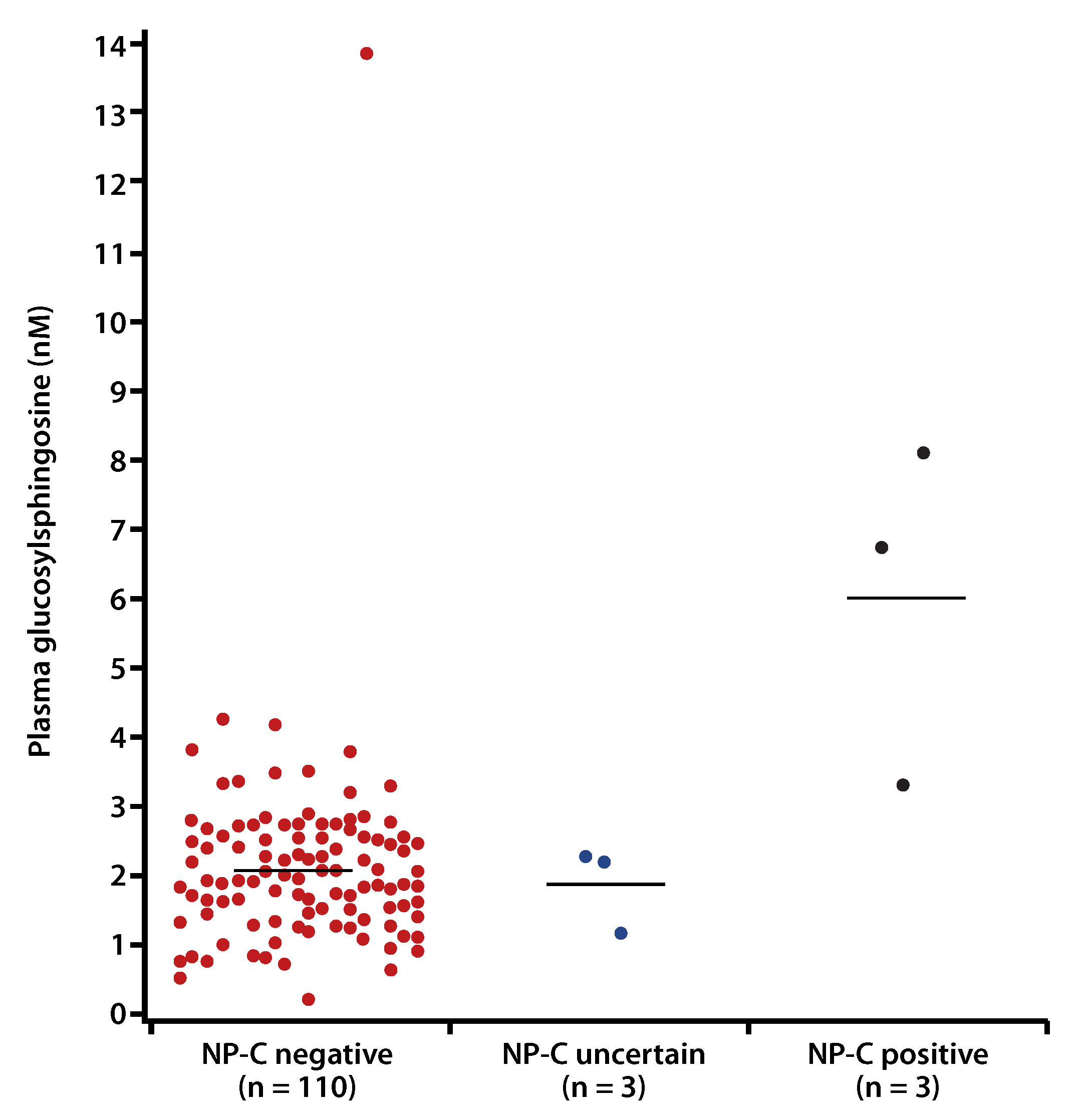

Supplement: Supplementary Data [file supp_ddt284_ddt284supp_fig2.tif]
